# A New Clinical Nomogram From the TCGA Database to Predict the Prognosis of Hepatocellular Carcinoma

**DOI:** 10.3389/fonc.2021.698980

**Published:** 2021-09-06

**Authors:** Dingde Ye, Jiamu Qu, Jian Wang, Guoqiang Li, Beicheng Sun, Qingxiang Xu

**Affiliations:** ^1^Medicine School of Southeast University Nanjing Drum Tower Hospital, Nanjing, China; ^2^Nanjing Medical University, Nanjing, China; ^3^Nanjing Drum Tower Hospital, The Affiliated Hospital of Nanjing University Medical School, Nanjing, China

**Keywords:** hepatocellular carcinoma, hepatectomy, nomogram, survival, prognosis

## Abstract

**Background and Aim:**

Hepatocellular carcinoma is a common malignant tumor of the digestive system with a poor prognosis. The high recurrence rate and metastasis after surgery reduce the survival time of patients. Therefore, assessing the overall survival of patients with hepatocellular carcinoma after hepatectomy is critical to clinicians’ clinical decision-making. Conventional hepatocellular carcinoma assessment systems (such as tumor lymph node metastasis and Barcelona clinical hepatocellular carcinoma) are obviously insufficient in assessing the overall survival rate of patients. This research is devoted to the development of nomogram assessment tools to assess the overall survival probability of patients undergoing liver resection.

**Methods:**

We collected the clinical and pathological information of 438 hepatocellular carcinoma patients undergoing surgery from The Cancer Genome Atlas (TCGA) database, then excluded 87 patients who did not meet inclusion criteria. Univariate and multivariate analyses were performed on patient characteristics and related pathological factors. Finally, we developed a nomogram model to predict patient’s prognosis.

**Results:**

A retrospective analysis of 438 consecutive samples from the TCGA database of patients with hepatocellular carcinoma who underwent potentially curative liver resections. Six risk factors were included in the final model. In the training set, the discriminative ability of the nomogram was very good (concordance index = 0.944), and the external verification method (concordance index = 0.962) was used for verification. At the same time, the internal and external calibration of the model was verified, showing that the model was well calibrated. The calibration between the evaluation of the nomogram and the actual observations was good. According to the patient’s risk factors, we determined the patient’s Kaplan-Meyer survival analysis curve. Finally, the clinical decision curve was used to compare the benefits of two different models in evaluating patients’ clinical outcomes.

**Conclusions:**

The nomogram can be used to evaluate the post-hepatectomy 1-, 3-, and 5-year survival rates of patients with hepatocellular carcinoma. The Kaplan-Meyer curve can intuitively display the survival differences among patients with various risk factors. The clinical decision curve is a good reference guide for clinical application.

## Introduction

Hepatocellular carcinoma (HCC) is the sixth most common malignant tumor in the world, and its mortality rate ranks fourth among malignant tumors (1). Hepatocellular carcinoma (HCC) accounts for 75-85% of primary liver cancer. The most important treatment in the early stage is hepatectomy (2–4). However, the high rate of recurrence and metastasis (60%) seriously affects the prognosis of patients, leading to low long-term survival rates of patients (2–4). In addition, the 5-year recurrence rate of patients is as high as 60-70% (5). Especially for patients with portal hypertension, the 5-year overall survival (OS) is only 37%. Tumor vascular invasion also seriously affects the survival rate of patients. This part of patients accounted for 18% (6).

Reducing postoperative recurrence is one of the most important measures to improve patient survival and prognosis. Therefore, accurate assessment of the prognosis of postoperative patients enables us to make correct clinical decisions in timely manner, thereby improving the prognosis and quality of life of patients. However, due to the complexity and heterogeneity of hepatocellular carcinoma, it is still a major challenge for clinicians to assess patient prognosis.

Due to the heterogeneity of hepatocellular carcinoma, it is difficult to evaluate the patient prognosis. In recent years, a variety of methods have been used to evaluate the prognosis, among which the nomogram has been widely used in clinical evaluation and improves prognosis assessment (7, 8). The nomogram is a graphical description of the predictive statistical model for a single patient (9). Although previous studies have evaluated the prognostic factors of patients with hepatocellular carcinoma (10), such as Barcelona clinical hepatocellular carcinoma (BCLC), tumor lymph node metastasis (TNM) and tumor markers (11), the evaluation is more cumbersome and it cannot calculate the specific survival probability. The nomogram is also an effective way to evaluate the prognosis of patients by integrating various factors (12). Its advantage is that it can refer to multiple factors at the same time and can intuitively display the prognosis of patients, which significantly improves the accuracy of prediction and clinical practicability. This study developed a nomogram combining multiple factors to predict the survival rate of patients after hepatocellular carcinoma surgery, so that clinicians can make better clinical decisions.

## Patients and Methods

### Patients

**Figure 1:**
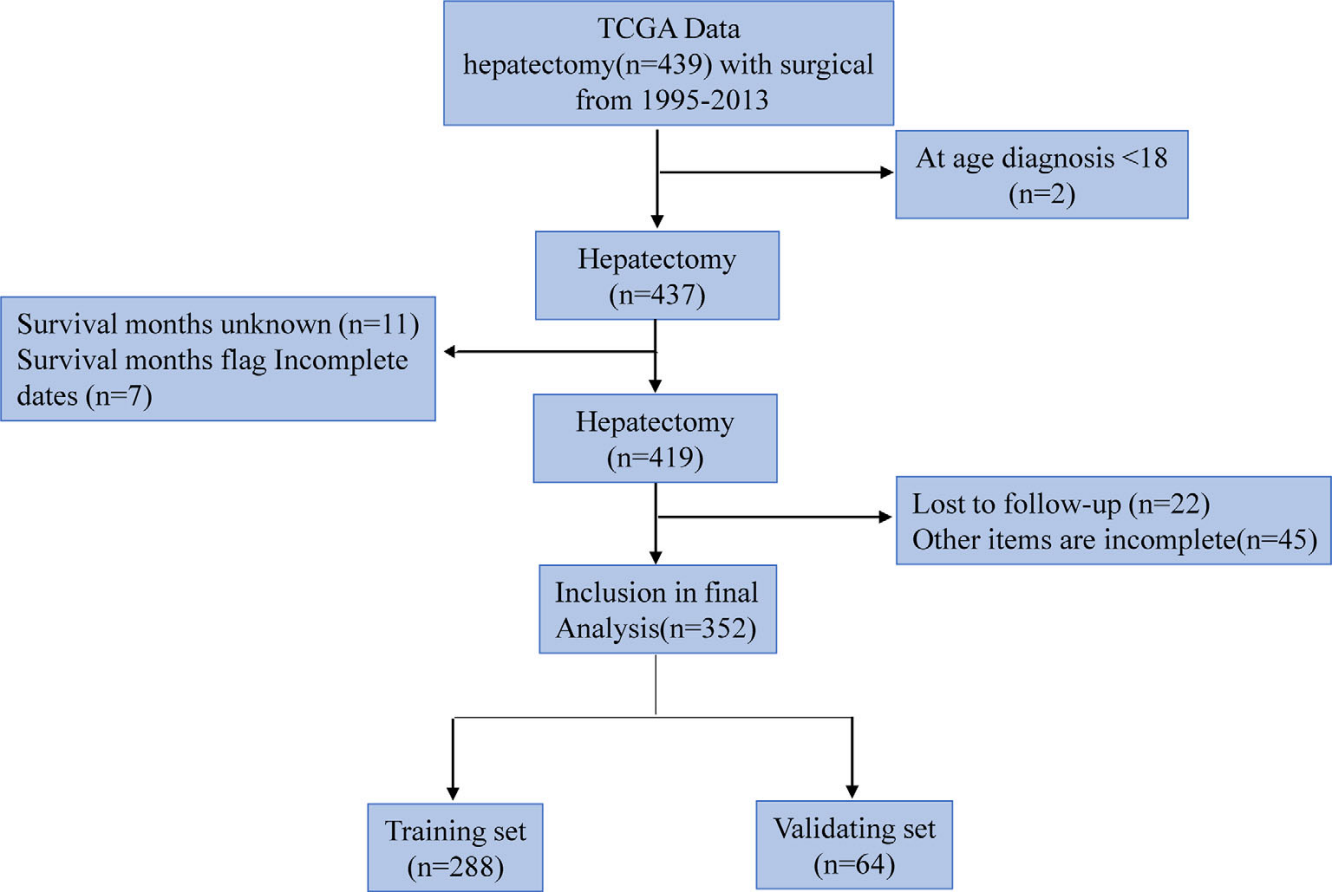
Numbers of patients enrolled and outcomes in the training set and validating set.

The research team retrospectively analyzed the clinical data of hepatocellular carcinoma patients extracted from the TCGA database. The TCGA project was jointly initiated by the National Cancer Institute (NCI) and the National Human Genome Research Institute (NHGRI) in 2006. At present, it has conducted research on a variety of cancer types, with the purpose of enhancing our understanding of the molecular basis of cancer, and further improving our ability to diagnose, treat and prevent cancer. We collected a total of 439 patients’ relevant information. Inclusion criteria: (1) hepatocellular carcinoma patients with surgical resection; (2) Age ≥18 years; (3) Complete clinical data. Exclusion criteria: (1) Age ≤18 years; (2) Incomplete clinical data; (3) lost to follow up. Of these, 87 patients were excluded, including 2 age Less than 18 year, 11 survival time unknow, 7 survival time flag incomplete dates, 22 lost to follow up, and 45 other items are incomplete, so 352 patients were finally included. Among them, 288 people were included in the training set and 184 cases survived; 64 people were included in the verification set and 36 cases survived. The selection and deletion process of patients is shown in **Figure 1**. Any information that can identify the patient’s identity is deleted. The report of this study is based on the Transparent Reporting of Multivariate Predictive Models for Individual Prognosis or Diagnosis (TRIPOD) reporting guidelines, which are used to establish models for multivariate diagnosis or predict patient prognosis to develop and validate disease diagnosis or prediction models (13). The nomogram predicts that the patient’s prognostic endpoint is the survival status within 1 year, 3 years and 5 years from the date of liver resection. According to the ratio of 9:2, the patients were randomly divided into training group and verification group. The prognosis yielded by the presumptive nomogram is based on prior clinical and pathological parameters related to the prognosis of hepatocellular carcinoma patients, including the degree of inflammation adjacent to the cancer (DFAC), gender, age, albumin (ALB), Child-Pugh score (Child-Pugh), creatinine value (CV), tumor histology grade (NHG), platelet count (PLT), vascular tumor cell type (VASTP), pathological stage (P. STAGE), body mass index (BMI), resection marginal residual tumor (RT), viral hepatitis serology (VHS), first-degree relative hepatocellular carcinoma (CFDR), total bilirubin (TB), AFP, risk factors (THCR), patient tumor status (PTS), postoperative treatment (POT), relative family cancer history (RFCH), TNM and other malignant tumors (OM).

### Statistical Analysis

In this study, continuous variables are represented by median and range. The categorical and continuous variables of the two groups were compared using the chi-square test and the unpaired Student’s t-test, respectively. Univariate and multivariate Cox regression were used to evaluate the risk factors related to the patient’s disease prognosis, and expressed in odds ratio (OR) and 95% confidence interval (CI). Variables (CV, P-STAGE, AFP, PTS, RT, TNM and VHS) with significant differences in univariate COX regression analysis were included in multivariate COX regression analysis to determine the final independent risk factors (CV, AFP, PTS, RT, TNM and VHS) for the patient’s prognosis. Finally, in order to predict the survival probability of HCC patients, through multivariate COX regression analysis with definite prognostic factors, we used the rms package of R software to draw a nomogram to predict the probability of overall survival at 1, 3, 5 years and median survival time after surgery. In order to verify the prognosis of HCC patients, the ROC curve was drawn using the survival ROC package of R software, and the difference between the model and different clinical indicators to evaluate the prognostic calibration of patients was compared. The overall survival time corresponding to each risk factor of the patient was calculated by the Kaplan-Meier method, and the Kaplan-Meier method was used to draw the survival curve. The performance of the model is evaluated by the calibration chart (to determine the agreement between the observed and estimated survival probabilities) and the verification index (to determine the discriminative ability of the model). Nomograms were constructed on the results of multivariate analysis using R software (US version 4.0.3). The prognostic nomogram was verified by the consistency index (C-index), receiver operating characteristic curve (ROC), decision curve analysis (DCA) and calibration curve. Statistical analysis was performed using IBM SPSS statistics 26.0 (IBM, Armonk, NY, USA) and MedCalc statistical software version 15.2.2 (MedCalc Software bvba, Ostend, Belgium; http://www.medcalc.org; 2015). P values below 0.05 are considered statistically significant.

## Results

### Patient Characteristics

A total of 439 HCC patients were extracted from the TCGA database. The characteristics of the target population can be seen in **Table 1**. 352 patients were finally included in the study, of which 288 patients entered the training set, and 64 cases were divided into the validation cohort. There were no significant differences in the characteristics of the two groups.

**Table 1:** Characteristics of patients with HCC in the training and validation group.

Characteristics	Total group (n = 352)		Training group (n = 288)		Validation group (n = 64)	
	N	%	N	%	N	%
**AGE**						
≤65	222	63	181	63	41	64
>65	130	37	107	37	23	36
**DFAC**						
0	228	65	187	65	41	64
1	104	29	83	29	21	33
2	20	6	18	6	2	3
**Child-Pugh**						
A	289	82	243	84	46	72
B	60	17	43	15	17	27
C	3	1	2	1	1	2
**CV**						
<50	14	4	13	5	1	2
50-100	232	66	187	65	45	70
>100	106	30	88	31	18	28
**NHG**						
1	40	11	35	12	5	8
2	173	49	132	46	41	64
3	126	36	109	38	17	27
4	13	4	12	4	1	2
**PLT**						
<100	51	14	40	14	11	17
100-300	281	80	231	80	50	78
>300	20	6	17	6	3	5
**VASTP**						
None	252	72	206	72	46	72
Micro	81	23	68	24	13	20
Macro	19	5	14	5	5	8
**P-STAGE**						
1	182	52	146	51	36	56
2	82	23	68	24	14	22
3	84	24	72	25	12	19
4	4	1	2	1	2	3
**BMI**						
<18.5	166	47	134	47	32	50
18.5-23.9	138	39	117	41	21	33
>23.9	48	14	37	13	11	17
**RT**						
R0	326	93	265	92	61	95
R1	9	3	9	3	0	0
R2	17	5	14	5	3	5
**VHS**						
None	187	53	152	53	35	55
HBV	76	22	60	21	16	25
HCV	18	5	15	5	3	5
HBV+HCV	71	20	61	21	10	16
**CFDR**						
None	248	70	206	72	42	66
Yes	104	30	82	28	22	34
**ALB**						
<3.5g/dl	43	12	34	12	9	14
>3.5g/dl	309	88	254	88	55	86
**TB**						
<1.4mg/dl	18	5	13	5	5	8
>1.4mg/dl	334	95	275	95	59	92
**AFP**						
≤25	105	30	88	31	17	27
25-400	159	45	134	47	25	39
>400	88	25	66	23	22	34
**THCR**						
None	318	90	260	90	58	91
Yes	34	10	28	10	6	9
**PTS**						
None	208	59	171	59	37	58
Yes	144	41	117	41	27	42
**POT**						
None	308	88	252	88	56	88
Yes	44	13	36	13	8	13
**RFCH**						
None	245	70	204	71	41	64
Yes	107	30	84	29	23	36
**Gender**						
Female	112	32	92	32	20	31
Male	240	68	196	68	44	69
**OM**						
None	181	51	145	50	36	56
Yes	171	49	143	50	28	44

DFAC, degree of inflammation adjacent to the cancer; Child-Pugh, Child pneumonia classification grade; CV, creatinine value; NHG, tumor histology grade; PLT, platelet count; VASTP, vascular tumor cell type; P.STAGE, pathological stage; BMI, body mass index; RT, resection marginal residual tumor; VHS, viral hepatitis serology; CFDR, first-degree relative hepatocellular carcinoma; ALB, albumin; TB, total bilirubin; AFP, alpha-fetoprotein; THCR, risk factors; PTS, patients Tumor status; POT, postoperative treatment; RFCH, relative family cancer history; OM, other malignant tumors.

### Independent Prognostic Factors for OS

Firstly, the univariate Cox regression analysis is used to determine the risk factors related to the patient’s prognosis, and then the univariate analysis risk factors are further incorporated into the multivariate COX regression analysis to determine the final independent risk factors for the patient’s prognosis. As shown in **Tables 2**, **3**, multivariate COX regression analysis shows that OS is significantly correlated with AFP, CV, PTS, RT, TNM and VHS.

**Table 2:** Univariable Cox regression model analyses of OS for nomogram.

Characteristics	OR	Univariable analysis		*p* value
		95% CI lower	95% CI upper	
**AGE**				
≤65y	reference			
>65y	1.308	0.884	1.934	0.181
**DFAC**				
0	reference			
1	0.640	0.390	1.050	0.079
2	0.657	0.286	1.510	0.325
**Child-Pugh**				
A	reference			
B	1.473	0.905	2.397	0.121
C	2.539	0.080	4.228	0.593
**CV**				
<50	reference			
50-100	<0.001	<0.0001	<0.0001	0.953
>100	0.650	0.433	0.976	0.038^**^
**NHG**				
1	reference			
2	1.220	0.650	2.288	0.538
3	1.224	0.640	2.342	0.544
4	1.350	0.434	4.197	0.606
**PLT**				
<100	reference			
100-300	1.405	0.809	2.439	0.229
>300	1.459	0.631	3.374	0.379
**VASTP**				
None	reference			
Micro	0.879	0.538	1.436	0.608
Macro	1.933	0.886	4.216	0.099
**P-STAGE**				
1	reference			
2	1.080	0.636	1.835	0.777
3	2.318	1.499	3.585	<0.001^**^
4	7.824	1.884	32.502	0.005^**^
**BMI**				
<18.5	reference			
18.5-23.9	0.976	0.654	1.458	0.906
>23.9	0.555	0.272	1.129	0.106
**RT**				
R0	reference			
R1	0.969	0.308	3.048	0.957
R2	2.791	1.211	6.430	0.017^**^
**VHS**				
None	reference			
HBV	0.607	0.367	1.004	0.053
HCV	1.205	0.578	2.515	0.620
HBV+HCV	1.873	1.107	3.169	0.020^**^
**CFDR**				
None	reference			
Yes	1.339	0.896	2.000	0.157
**ALB**				
<3.5g/dl	reference			
>3.5g/dl	1.435	0.725	2.840	0.303
**TB**				
<1.4mg/dl	reference			
>1.4mg/dl	1.066	0.435	2.613	0.889
**AFP**				
≤25	reference			
25-400	1.289	0.724	2.293	0.391
>400	3.646	2.485	5.351	<0.001^**^
**THCR**				
None	reference			
Yes	0.919	0.478	1.768	0.802
**PTS**				
None	reference			
Yes	2.129	1.436	3.158	<0.001^**^
**POT**				
None	reference			
Yes	1.180	0.701	1.988	0.535
**RFCH**				
None	reference			
Yes	1.351	0.907	2.014	0.141
**Gender**				
Female	reference			
Male	1.044	0.695	1.569	0.838
**OM**				
None	reference			
Yes	1.279	0.861	1.898	0.225
**TNM**				
I	reference			
II	2.200	1.374	3.521	0.001^**^
III	2.871	1.761	4.682	<0.001^**^
IV	3.237	1.272	8.238	0.014^**^

OR, Odds ratio; CI, confidence interval; **, It is statistically significant and included in the multivariate COX regression analysis. DFAC, degree of inflammation adjacent to the cancer; Child-Pugh, Child pneumonia classification grade; CV, creatinine value; NHG, tumor histology grade; PLT, platelet count; VASTP, vascular tumor cell type; P.STAGE, pathological stage; BMI, body mass index; RT, resection marginal residual tumor; VHS, viral hepatitis serology; CFDR, first-degree relative hepatocellular carcinoma; ALB, albumin; TB, total bilirubin; AFP, alpha-fetoprotein; THCR, risk factors; PTS, patients Tumor status; POT, postoperative treatment; RFCH, relative family cancer history; OM, other malignant tumors, TNM, tumor node metastasis.

**Table 3:** Multivariable Cox regression model analyses of OS for nomogram.

Characteristics	OR	Multivariable		*p* value
		95% CI (lower)	95% CI (upper)	
**AFP**				
≤25	Reference			
25-400	2.626	1.742	3.958	<0.001
>400	3.742	2.505	5.590	<0.001
**CV**				
≤50	Reference			
>100	1.802	1.155	2.812	0.010
**PTS**				
None	Reference			
Yes	2.012	1.320	3.065	0.001
**RT**				
R0	Reference			
R2	6.731	2.759	16.424	<0.001
**VHS**				
None	Reference			
HBV+HCV	2.245	1.426	3.535	0.001
**TNM**				
I	Reference			
II	2.679	1.655	4.335	<0.001
III	3.724	2.244	6.181	<0.001

OR, Odds ratio; CI, confidence interval; AFP, alpha-fetoprotein; CV, creatinine value; PTS, patients Tumor status; RT, resection marginal residual tumor; VHS, viral hepatitis serology; TNM, tumor node metastasis.

### Generation of the Prognostic Nomogram

**Figure 2:**
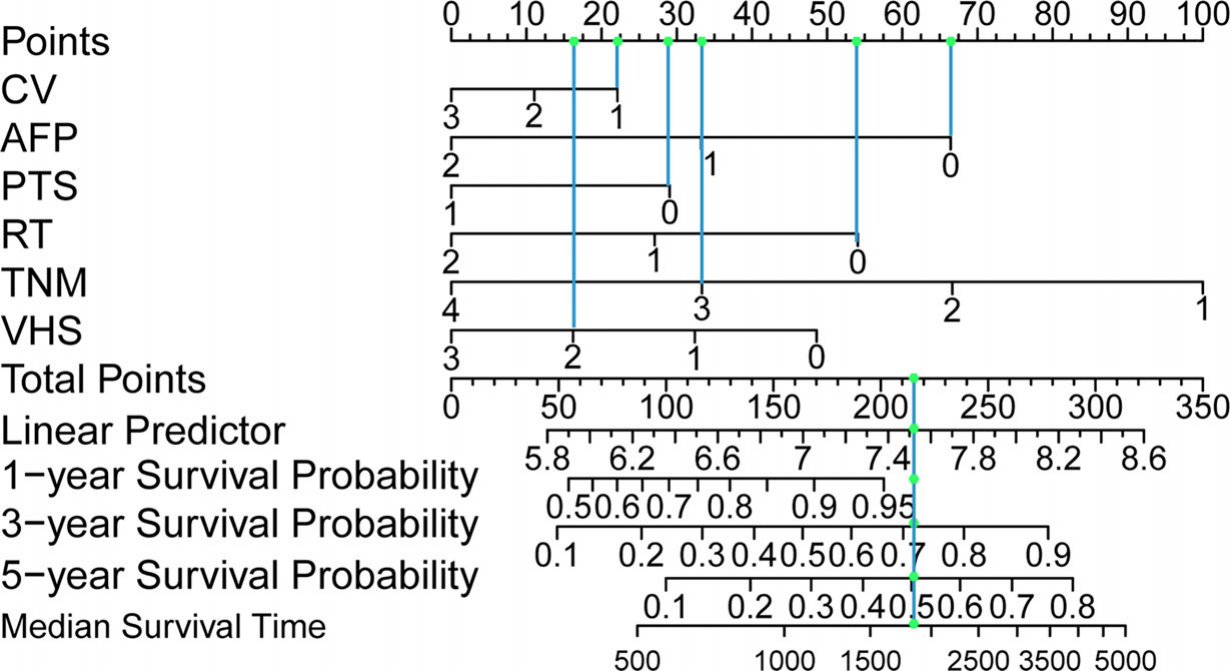
Postoperative hepatocellular carcinoma prognostic nomogram. (To use the nomogram, a patient’s values are located on each variable axis, and a line is drawn upward to determine the number of points received for each variable value. The sum of these numbers is located on the total point axis, and a line is drawn downward to the survival axes to determine the probability of survival at 1, 3 and 5 years and the median survival time.). CV, creatinine value; AFP, alpha-fetoprotein; PTS, patients Tumor status; RT, resection marginal residual tumor; TNM, tumor node metastasis; VHS, viral hepatitis serology.

Based on multivariate analysis, we generated a nomogram for six independent risk factors (**Figure 2**). According to the independent risk factors of the patient, a relevant score is assigned to each independent risk factor, and a total score is finally obtained. The vertical line generated by the total score helps predict the 1-year, 3-year, or 5-year survival rate, and the median survival time can be predicted after the total score is calculated. Determining the total score can enable us to more accurately and easily estimate the survival probability and median survival time of patients, and timely intervene independent risk factors that affect the survival and prognosis of patients. The graph visually shows the relative probability of overall survival for patients with relevant risk factors.

### Predictive Ability of the Nomogram Model

**Figure 3:**
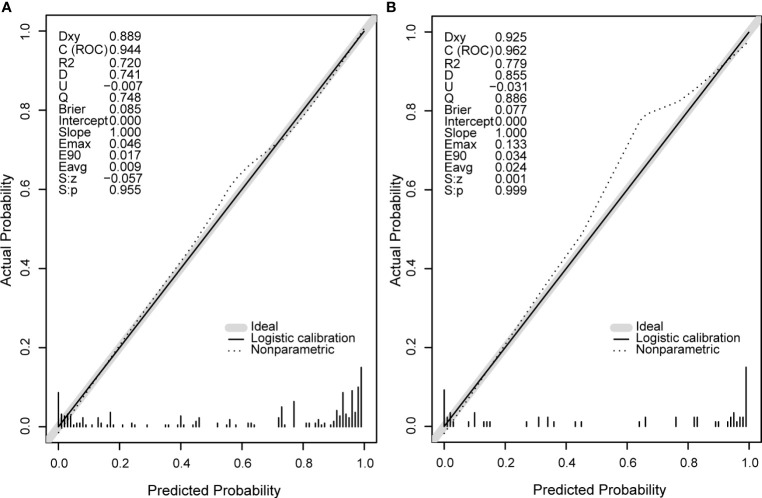
The calibration curve diagrams of the training set **(A)** and the validation set **(B)** have good agreement between the predicted probability and the actual probability, both S, p > 0.05. Emax, the maximum offset between the model and the ideal model; Eavg, the minimum offset between the model and the ideal model.

**Figure 4:**
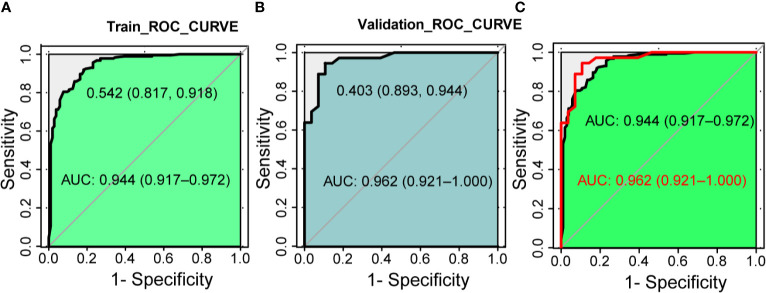
The discrimination test of nomogram model (ROC analysis). **(A)** The area under the AUC of the training set is 0.944(95%CI 0.917~0.972, P < 0.001), the best diagnostic threshold is 0.542. **(B)** The AUC of the validation set is 0.962 (95% CI 0.921~1.000, P < 0.001), the best diagnostic cut-off value is 0.403. **(C)** Combination of panels **(A, B)**. AUC, the area under the receiver operating characteristic curve; ROC, the receiver operating characteristic.

Next, we compare the calibration and the discrimination inside and outside the model. The degree of calibration is better displayed in **Figure 3**, and the *P* value of both is greater than 0.05. The degree of discrimination is represented by the area under the receiver operating characteristic curve (AUROC). The larger the area under the curve, the better the degree of discrimination. Generally, AUROC > 0.6 indicates that the model has a good discrimination. The AUROC of the training set was 0.944 (95% CI: 0.917-0.972), the cutoff value was 0.542 (*P* < 0.0001), and the C index was 0.944. The AUROC of the verification set was 0.962 (95% CI: 0.921-1.000) (*P* < 0.0001) and the C index was 0.962. The C index of the prediction model in both populations was > 0.75 and showed a good degree of discrimination as shown in **Figure 4**.

### Validation of the Prognostic Nomogram

**Figure 5:**
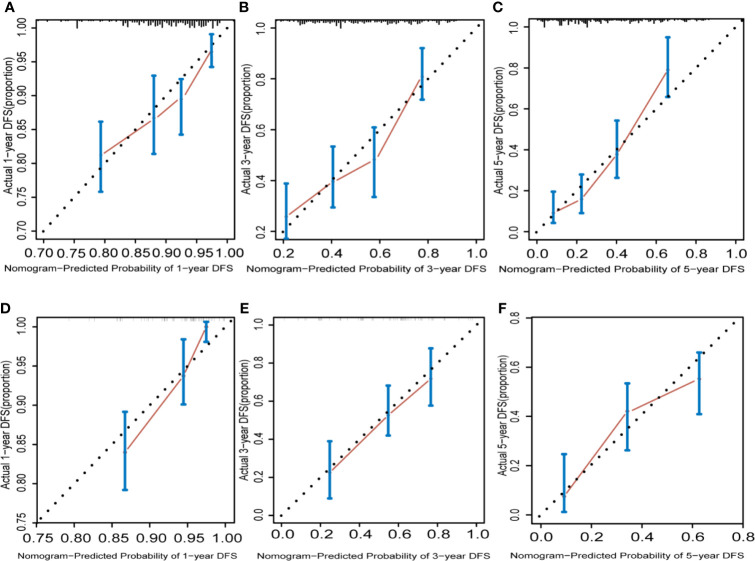
Hepatocellular carcinoma calibration curve. The calibration curve for predicting OS at 1 years **(A)** for training cohort; **(D)** for validation cohort), 3 years **(B)** for training cohort; **(E)** for validation cohort) and 5 years **(C)** for training cohort; **(F)** for validation cohort) in HCC patients. Nomogram-predicted probability of overall survival is plotted on the x axis and actual overall survival is plotted on the y axis.

The calibration graphs for internal verification and external verification are basically linear, showing excellent agreement between the estimated values of the nomogram and the actual observations in the 1-year, 3-year, and 5-year survival probabilities (**Figure 5**). In addition, all the prediction lines overlap the reference lines well, which proves the good performance of the nomogram.

### Decision Curve Analysis

**Figure 6:**
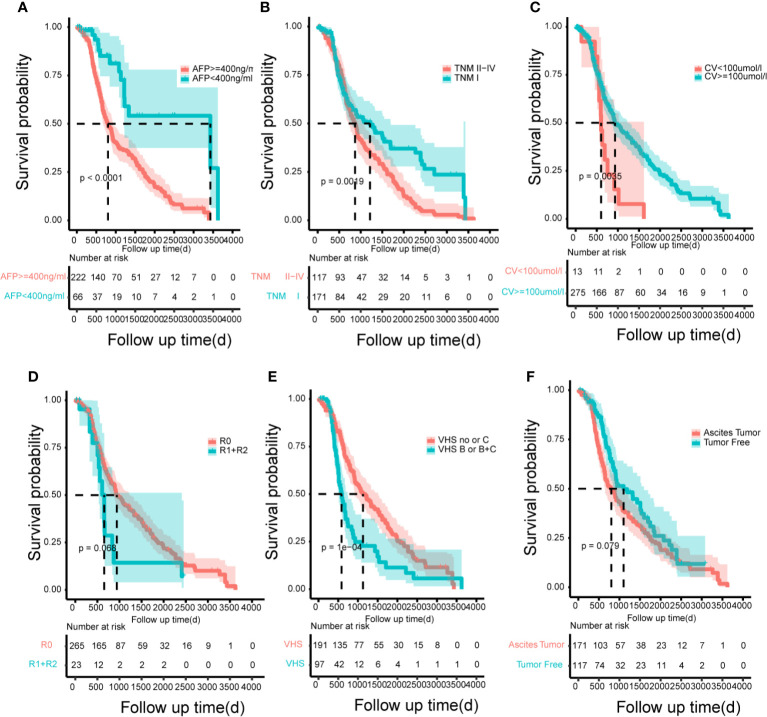
The Kaplan–Meier curves for subgroups of patients. Patients were stratified by the AFP **(A)**, tumor node metastasis **(B)**, creatinine value **(C)**, resection marginal residual tumor **(D)**, viral hepatitis serology **(E)** and patient’s tumor status **(F)** in the training cohort.

According to the risk factors of the model, in order to emphasize the control of prognostic factors, we finally drew the Kaplan-Meyer survival curves (**Figure 6**) to show the benefit of treatment based on the total population in this study. The risk factors showed significant statistical differences.

**Figure 7:**
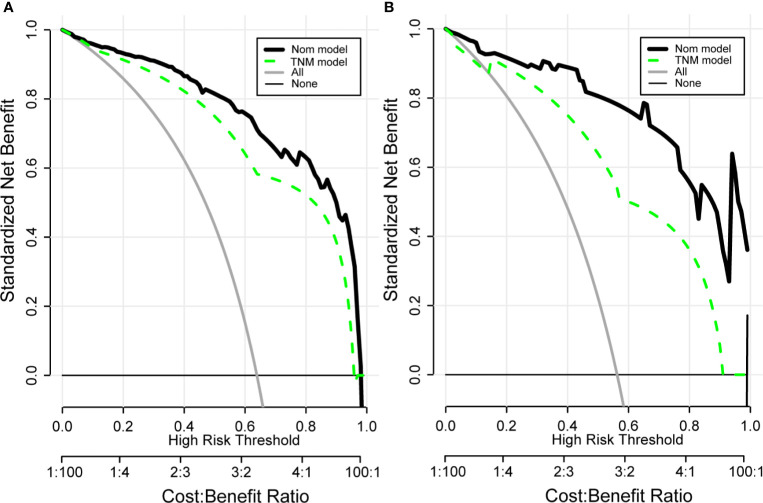
Clinical decision curve analysis (DCA) of the training group **(A)** and validation group **(B)** for clinical benefits. Comparison of DCA between Nom model and TNM model. X-axis stands for threshold probability. Y-axis stands for the net benefit after the advantage minus the disadvantage. The black horizontal line indicates that all samples are negative, which means none of the patients received interventions and the net benefit is 0; The gray oblique line indicates that all samples are positive and all patients received interventions. Nom model included AFP, VHS, CV, RT, TNM, PTS. TNM Model included T-stage, N-stage, and M-stage.

Drawing a decision curve analysis (DCA) can visually show the clinical benefits of patients. The DCA curve we drew shows that in the training and validation groups, our nomogram has a positive net benefit to patients, and has a wider range of benefit probability (**Figure 7**). And it can be seen that compared with the TNM prognostic evaluation system, the nomogram model has greater clinical net benefits and has obvious advantages in prognostic evaluation.

## Discussion

Previous studies have shown that in tumor prognosis prediction models, nomograms are more practical and accurate than tumor staging systems (14–16). Based on this, we generated and verified the nomogram model which combines six independent risk factors for the prognosis assessment of hepatocellular carcinoma patients to accurately predict the survival rate of HCC patients. The calibration curve shows that there is a high degree of consistency between the predicted value and the actual value in the training set and the verification set, which confirms that the column graph model has good repeatability and provides a reliable reference basis for clinicians’ clinical decision-making. At the same time, in the 1-year, 3-year and 5-year survival rates, the nomogram evaluation model predicts OS better than other commonly used evaluation systems. In addition, in order to evaluate the clinical benefit obtained by the patient through the nomogram, we visually display it through the DCA curve. DCA is usually used to evaluate the maximum clinical net benefit of the model (17, 18), and is generally considered to be more accurate than the ROC curve (19). The net benefit of our nomogram model for clinical decision-making is significantly better than the TNM prognostic evaluation system, and it has been verified in the validation set. These results represent an excellent estimate of the outcome of the decision at a higher threshold probability level.

Although clinical guidelines provide clinical indications for treatment, we pay more attention to the prognosis of patients after surgery. Moreover, clinicians lack effective predictive methods (20), making them likely to be unable to predict the patient’s prognosis and miss the best time for end-of-life discussions and/or end-of-life care referrals (21). In addition, studies have shown that many patients were eager to understand their prognosis after hepatectomy (22). Therefore, this study explored the survival 1, 3, and 5 years after hepatectomy. Our nomogram can not only help clinicians make key treatment decisions but also provide patients with very important survival information based on individual risk factors.

Our nomogram involved six independent risk factors. These factors have already been shown to correlate with poor prognosis after HCC resection (23–27). In terms of these risk factors, our research is consistent with previous studies.

Tumor differentiation is related to the prognosis of liver tumors. A poor degree of differentiation can lead to poor prognosis. Previous studies have shown that the prognosis of hepatocellular carcinoma is related to poorer histopathological grades (28).

The accurate risk stratification of patients with postoperative hepatocellular carcinoma prognosis is critical, as the prognosis of patients may vary (29). Comprehensive consideration of a variety of factors will be more helpful for postoperative prognosis assessment of patients, and our nomogram model shows that the internal and external predictions (C-index: 0.944 and 0.962) of the model are good, suggesting that our nomogram can be better predict the survival rate of patients with hepatocellular carcinoma after surgery.

In summary, our nomogram performed well in internal verification and external verification. This study also has certain limitations. First of all, the study is a retrospective study. The data comes from a single hepatocellular carcinoma database, and there may be corresponding errors in the accuracy of the data. The sample size is still small, and more research is needed to verify the established nomogram from the outside. In addition, because the database lacks imaging data to evaluate tumor characteristics, the nomogram model is temporarily unable to evaluate its potential impact.

## Conclusion

Above all, we have developed and verified a nomogram that predicts the 1-year, 3-year, and 5-year survival rates of HCC patients based on a large number of population-based cohorts. The nomogram prediction model shows higher prediction accuracy than the TNM staging system. Therefore, through this model, clinicians can more accurately estimate the survival rate of a single patient, and promptly intervene in the risk factors of high-risk patients to improve the patient’s prognosis.

## Data Availability Statement

Publicly available datasets were analyzed in this study. This data can be found here: TCGA database.

## Ethics Statement

The studies involving human participants were reviewed and approved by Nanjing Drum Tower Hospital, The Affiliated Hospital of Nanjing University Medical School.

## Author Contributions

DY, QX, and JQ designed the study. DY and JW analyzed the data and wrote the manuscript. DY, QX, GL, and BS provided technical expertise and support. All authors contributed to the article and approved the submitted version.

## Conflict of Interest

The authors declare that the research was conducted in the absence of any commercial or financial relationships that could be construed as a potential conflict of interest.

## Publisher’s Note

All claims expressed in this article are solely those of the authors and do not necessarily represent those of their affiliated organizations, or those of the publisher, the editors and the reviewers. Any product that may be evaluated in this article, or claim that may be made by its manufacturer, is not guaranteed or endorsed by the publisher.
